# Supplementary material for: Identification of photoperiod-induced specific miRNAs in the adrenal glands of Sunite sheep (Ovis aries)
Source: Front Vet Sci. 2022 Jul 22;9:888207. doi: 10.3389/fvets.2022.888207 (PMC9354845; doi:10.3389/fvets.2022.888207)
Supplement: Supplementary file 1 [file Data_Sheet_1.ZIP › Supplementary file/miRDeep2 file.zip!/miRDeep2 file/Novel_Structure/LP42A1/Novel_195.pdf]

Provisional ID	:	NC_019476.2_28165
Score total	:	2.5
Score for star read(s)	:	-1.3
Score for read counts	:	0
Score for mfe	:	2.2
Score for randfold	:	1.6
Score for cons. seed	:	
Total read count	:	125
Mature read count	:	123
Loop read count	:	0
Star read count	:	2

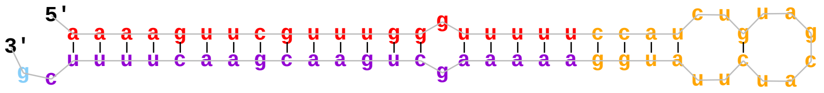

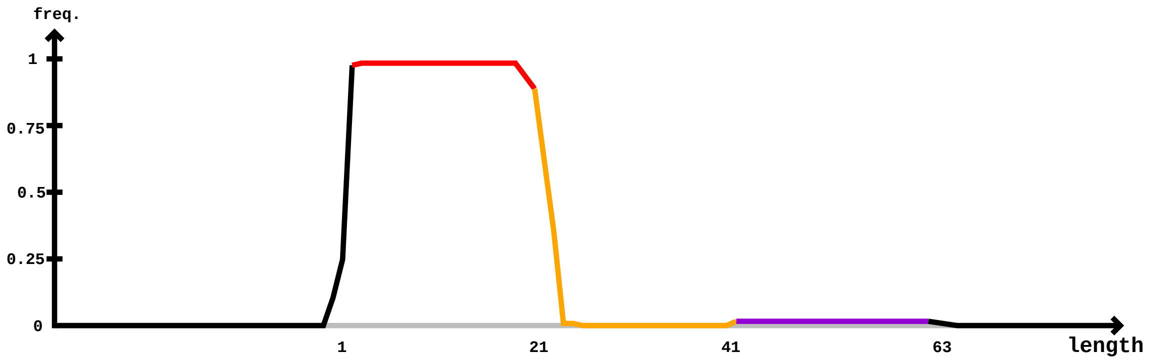

Mature **Star**

```
5'- guccacaagucuuuguauuagguuggcccaaaaguucguuuggguuuuuccaucuguagcaucuuauggaaaaagcugaacgaacuuuucggccaauccaguaugagga   -3'   obs
    guccacaagucuuuguauuagguuggcccaaaaguucguuuggguuuuuccaucuguagcaucuuauggaaaaagcugaacgaacuuuucggccaauccaguaugagga         exp
    .........((((((((((.((((((((.((((((((((((((.(((((((((..(......)..))))))))).)))))))))))))).)))))))).))))))))))   reads   mm   sample
    ...........................cAaaaaguucguuuggguuuu.............................................................   5       1    seq
    ...........................cAaaaaguucguuuggguuuuu............................................................   7       1    seq
    ...........................cAaaaaguucguuuggguuuuuc...........................................................   1       1    seq
    ............................Aaaaaguucguuuggguuu..............................................................   6       1    seq
    ............................caaaaguucguuuggguuuu.............................................................   1       0    seq
    ............................Gaaaaguucguuuggguuuuu............................................................   9       1    seq
    ............................caaaaguucguuuggguuuuu............................................................   1       0    seq
    ............................Gaaaaguucguuuggguuuuuc...........................................................   1       1    seq
    .............................aaaaguucguuuggguuuuu............................................................   29      0    seq
    .............................aaaaguucguuuggguuuuC............................................................   6       1    seq
    .............................aaaaguucguuuggguuuuuA...........................................................   6       1    seq
    .............................Gaaaguucguuuggguuuuuc...........................................................   1       1    seq
    .............................aaaaguucguuuggguuuuuU...........................................................   6       1    seq
    .............................aaaaguucguuuggguuuuucU..........................................................   11      1    seq
    .............................Gaaaguucguuuggguuuuucc..........................................................   1       1    seq
    .............................aaaaguucguuuggguuuuucA..........................................................   28      1    seq
    .............................aaaaguucguuuggguuuuucG..........................................................   3       1    seq
    ..............................aaaguucguuuggguuuuuccGu........................................................   1       1    seq
    .....................................................................aaaaaCcugaacgaacuuuuc...................   2       1    seq
```
